# High performance of Mn-Co-Ni-O spinel nanofilms sputtered from acetate precursors

**DOI:** 10.1038/srep10899

**Published:** 2015-06-08

**Authors:** Zhiming Huang, Wei Zhou, Cheng Ouyang, Jing Wu, Fei Zhang, Jingguo Huang, Yanqing Gao, Junhao Chu

**Affiliations:** 1 National Laboratory for Infrared Physics, Shanghai Institute of Technical Physics, Chinese Academy of Sciences, 500 Yu Tian Road, Shanghai 200083, Peoples Republic of China; 2 Key laboratory of Space Active Opto-Electronics Technology, Shanghai Institute of Technical Physics, Chinese Academy of Sciences, 500 Yu Tian Road, Shanghai 200083, Peoples Republic of China

## Abstract

Mn-Co-Ni-O (MCN) spinel oxide material, a very important transition metal oxide (TMO) with the best application prospects in information and energy fields, was discovered over five decades ago, but its applications have been impeded by the quality of its films due to the magnitude of deposition challenge. Here we report that high quality of MCN nanofilms can be achieved by sputtering deposition via acetate precursors whose decomposition temperatures are matched to the initial synthesis temperature of the MCN thin films. Excellent performance of MCN nanofilms is demonstrated, combining for the first time preferred orientation, high temperature coefficient of resistance, and moderate resistivity. The film devices show an intrinsic recombination with a much faster rate of the order of a microsecond for the laser-pumped carriers, which is ~3 orders of magnitude larger compared with that of the ceramic material. The electronic structure of the thin films confirms that it is indeed of n-type nature, exhibiting appropriate electronic states consistent with the levels of metal electrodes and semiconductors. The results offer a vital avenue for depositing high performance TMO thin films for advanced oxide devices, and will have great significance for exploiting new applications in modern oxide electronics and optoelectronics.

Nowadays transition metal oxides (TMO) play an increasingly important role in a variety of novel applications due to their exceptional electronic properties. TMO are utilized in a variety of applications, such as high-temperature superconductivity^1^, multiferroics^2^,^3^, solar cell^4^, oxide electronics^5^, organic electronics^6^, photodetectors^7^ and thermistors^8^, which result from a combination of the strong correlation between the localized transition metal valence electrons and the strongly polarizable metal-oxygen bond. However, the quality of TMO depends strongly on the process of the sample preparation^9^,^10^.

Among them, Mn-Co-Ni-O (MCN) spinel oxide is considered to be the most important TMO material with extensive application prospects in the fields of information and energy^11^. The high polarizability between the transition metals Mn, Co, and Ni and oxides causes a strong coupling to the external electromagnetic wave, which produces a very broadband spectral response (0.2–50 μm). The strong electron correlations in metal Mn produce semiconducting character with moderate resistivity (~10–10^3^ Ω·cm)^12^ compatible with integrated circuits and extremely high absolute temperature coefficient of resistance (TCR ~ −4% K^−1^) at room temperature. All those features make MCN thin films play an important role in oxide electronics and optoelectronics. Unfortunately, the strong electron correlations have a significant impact on the deposition quality of the MCN films. Although this ceramic material was discovered over 50 years ago, there are still no reports of realizing high performance nanoscale thin films, which restricts its prospects of successful application.

In this paper, we report the growth of highly oriented (220) MCN spinel nanofilms from suitable precursors whose decomposition temperatures are matched to the initial synthesis temperature of the MCN films during fabrication procedure. High quality MCN nanoscale films are achieved combining for the first time excellent performance of TCR and moderate resistivity. The film devices show a response time ~3 orders magnitude shorter than that of the ceramic material devices for laser-pumped carrier recombination and suitable electronic structure matched to metals and semiconductors.

MCN spinel oxide develops from the precursor Mn_3_O_4_. It has been widely used in bolometer and uncooled infrared detection because of its excellent negative temperature coefficient of resistance (TCR) characteristics and long-time stability^8^. MCN ceramic materials sintered at high temperature of over 1000 °C have been employed for a long time in mature applications. With the development of film technology, MCN thin films have been fabricated by utilizing membrane techniques of chemical solution deposition (CSD)^13^, screen printing method^14^, and radio frequency (RF) sputtering^15^,^16^,^17^. The performance of MCN, similarly to other TMO materials, is strongly related to the oxidation states of cations. This is because its transport is dominated by complex percolation theory of small polaron hopping conduction, and its quality is very sensitive to the oxidation ratio of Mn^3+^ and Mn^4+^ ions distributed over octahedral sites in the spinel cubic structure. To the best of our knowledge, there has been no breakthrough in the deposition of the high-quality nanometric films. Compared to other synthesis routes, such as CSD and screen printing, RF magnetron sputtering method is much more precise in controlling growth conditions, and highly efficient, as well as more suitable for large scale applications^14^. In the present paper we have chosen to use the RF sputtering method which has been utilized to fabricate MCN thin films for over two decades^18^. However, previous works failed to produce films of high quality with respect to crystallinity and structural properties^15^,^17^,^19^.

As shown in Fig. 1, previous RF targets were mainly made from raw materials of nitrates, carbonates or metallic oxides. They are either unstable in the atmosphere, or their decomposition temperatures (see Table S1, Supporting Information) differ greatly from that of the initial crystallization temperature of MCN thin films (begins at about 400 °C)^20^, which gives rise to oxidation states of Mn^2+^ ions occupied at tetrahedral sites and of Mn^3+^ and Mn^4+^ ions distributed at octahedral sites in the spinel structure. As a consequence, high quality nanoscale MCN films are still lacking, and there is at present no solution for growing MCN nanofilms for extensive potential applications^11^. Here we use acetates of Mn, Co and Ni to synthesize the RF target material for MCN, because the end temperatures of the acetate decomposition reaction are much closer to the starting temperature of the MCN crystallization reaction. That is, the decomposition of the precursors is matched to the crystallization process of MCN thin films, which promises an accurate manipulation of the oxidation states of Mn, Co and Ni ions in the spinel structure.

## Results and Discussion

**Figure 2:**
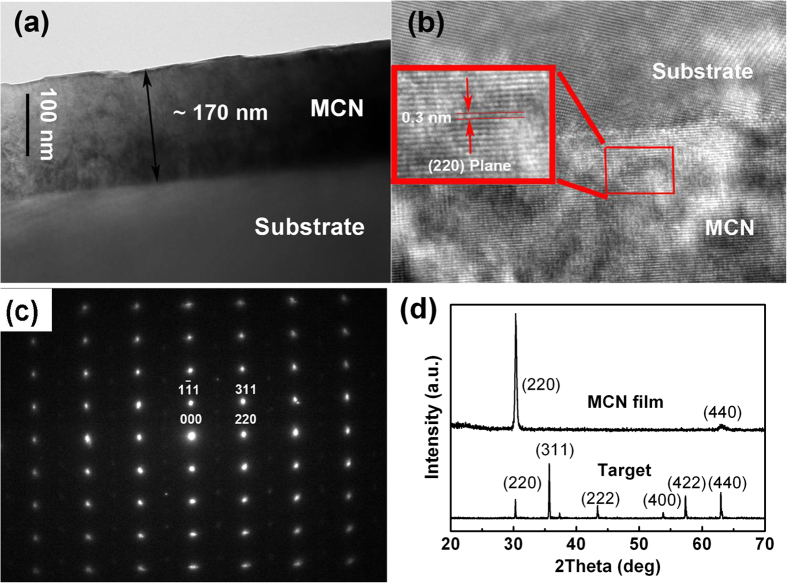
Structures of the MCN nanofilms on Al_2_O_3_ substrate. (**a**) Thickness of the MCN measured by cross sectional TEM image. (**b**) HRTEM image taken at the interface of MCN film and substrate. (**c**) Electron diffraction pattern of the MCN films in the 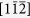 zone axis. (**d**) XRD spectra of the MCN nanofilms and the corresponding target material.

MCN nanofilms with nominal composition of Mn_1.4_Co_1.0_Ni_0.6_O_4_ are deposited by the target material, sintered from the stoichiometric acetate precursors of Mn(CH_3_COO)_2_·4 H_2_O, Co(CH_3_COO)_2_·4H_2_O, Ni(CH_3_COO)_2_·4H_2_O with an atomic ratio of 7:5:3. The thickness of the films is about 170 nm, measured by cross sectional TEM image (Fig. 2a). The lattice fringes of (220) planes are clearly observed by HRTEM image with the distance of 0.3 nm in MCN films, as shown in Fig. 2b. The selected area of electron diffraction (SAED) patterns of the 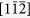 zone in Fig. 2c further support the excellent crystallization of the MCN films. The XRD patterns in Fig. 2d shows that sharp (220), (440) peaks are formed. It means that the MCN films possess high quality crystallization with good orientation, which differs greatly from that of the target material and former reports on MCN material. The diffraction peaks have been indexed into a spinel cubic structure with the lattice constant of a = 0.831 ± 0.001 nm (Referenced to NiMn_2_O_4_ with the JCPDS Card No. 84 – 0542).

**Figure 3:**
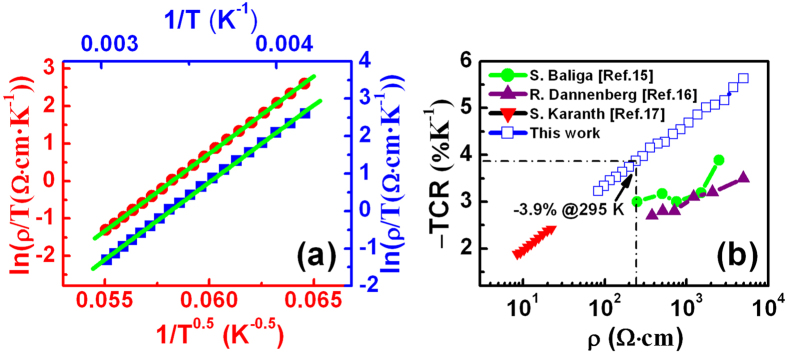
Electrical properties of the MCN nanofilms. (**a**) Plots of ln(*ρ/T*) vs *1/T *^0.5^ (red color) and ln(*ρ/T*) vs *1/T* (blue color) and the corresponding linear fittings (green color). (**b**) Comparison of negative TCR values as a function of resistivity in this work and from that in Refs. 15, 16, 17.

To investigate the electrical properties of the MCN nanofilms, the temperature dependent resistivity is given by the expression^21^:


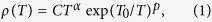


where 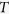 is the absolute temperature, 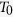 is the characteristic temperature. Figure 3a shows the ln(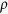/*T*) − *1*/*T*^0.5^ plot for variable range hopping (VRH) with 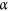 = 2*p*. A good linear fitting relationship exists, which implies that the conduction is dominated by VRH model with a parabolic shaped density of states (DOS) around the Fermi level. To derive the material constant B of MCN, Fig. 3a also shows the ln(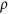
*/T)* − *1/T* plot, we assumed approximately nearest neighbor hopping (NNH) with 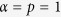. The activation energy of E_0_ ≈ 0.296 eV is derived by applying the formula E_0_ = *k*_B_B (where *k*_B_ is Boltzmann constant). The advantage of MCN ceramic materials is that they have a high TCR≈–3.50%K^–1^ and a moderate resistivity of 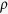 ≈ 250 Ω·cm at room temperature. Nevertheless, these values were not achieved by RF sputtering of MCN films in former reports^15^,^16^,^17^. We have realized the first MCN nanofilms possessing simultaneously moderate resistivity of ~250 Ω·cm and high TCR value of ~ –3.9% K^−1^, at temperature of 295 K (Fig. 3b), which enables small temperature variations caused by absorbed infrared radiation to generate a significant voltage drop across the bolometer and make MCN nanofilms suitable for thermometer and infrared sensing applications.

The conduction mechanism of MCN is small polaron hopping between mixed valence of Mn^3+^ and Mn^4+^, with the assistance of thermal activation, which contributes to the electrical conduction. According to the Nernst–Einstein equation, the temperature dependent resistivity 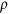 of MCN films is described in the form^22^:


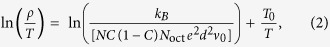


where 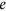 is electronic charge, 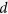 is hopping distance, 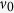 is hopping frequency, and *N*_oct_ is the concentration of octahedral sites. The factor 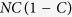 denotes the probability that adjacent sites will be occupied by a Mn^3+^ and Mn^4+^ redox couple. Considering a fixed 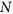 value, the 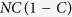 is maximized when [Mn^3+^]_oct_ = [Mn^4+^]_oct_^23^. To verify the optimal oxide states in the MCN films, we have investigated the XPS spectra for Mn 2p^3/2^ signals of the MCN films.

**Figure 4:**
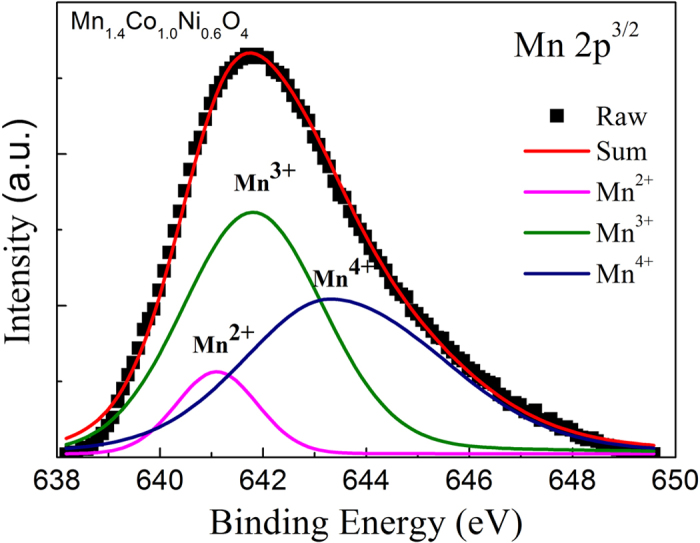
Mn 2p^3/2^ XPS spectrum and deconvoluted curves for Mn^2+^, Mn^3+^ and Mn^4+^ after background subtraction.

The XPS spectrum for Mn 2p^3/2^ signals of the MCN nanofilms can be deconvoluted, after background subtraction by a fitting process, using XPS standard software, where the binding energies and FWHM values of Mn^2+^, Mn^3+^ and Mn^4+^ are referenced by MnO, ZnMn_2_O_4_ and MgNiMnO_4_ compounds, respectively^24^. As shown in Fig. 4, the Mn 2p^3/2^ signal is fitted to optimize the intensities of the three peaks belonging to the Mn^2+^, Mn^3+^ and Mn^4+^ oxidation states after a least square procedure. A Mn cation distribution in our MCN spinel structure can be expressed as [Mn^2+^]:[Mn^3+^]:[Mn^4+^] = 0.118:0.641:0.641, which verifies that the composition of Mn^3+^ matches well with that of Mn^4+^. It shows that the optimal electrical conductivity is reached only when the concentration of [Mn^3+^]_oct_ is equal to that of [Mn^4+^]_oct_ (see Figure S1 in Supporting Information).

The outstanding detection ability is evaluated for potential infrared bolometer in Figure S2 of Supporting Information. The great improvement in the estimated performance is attributed to the optimized oxidizing status and grain orientation properties of the MCN films, which have benefited from the improved cationic manipulation of the target material manufactured from acetate. In addition, the films show great potential for development in focal plane array detection.

To find a wide range of potential applications for MCN, it is critical to determine unambiguously the electronic structure of the films, including the conduction type, work function (WF), ionization energy (IE), and electron affinity (EA). Ultra-violet photoemission spectroscopy (UPS) is a widely used technique that provides direct measurement of the density of electronically filled states of materials. Combined with the determination of band gap energy of the materials by optical method, the valence band (VB) and conduction band (CB) states of the films can be established.

**Figure 5:**
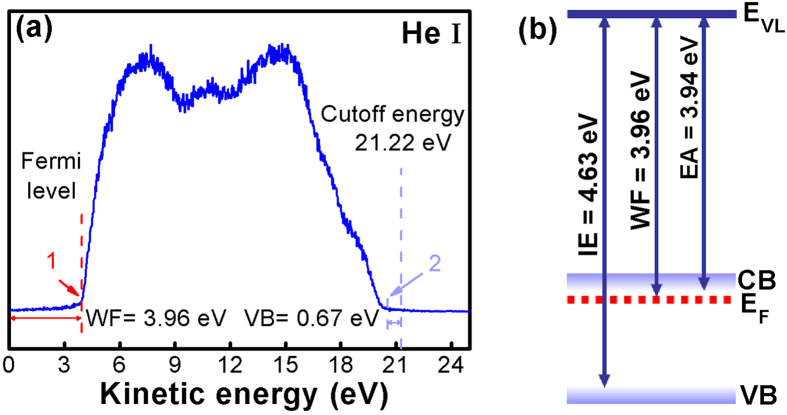
(**a**) UPS spectrum of the MCN nanofilms. The marks of positions 1 and 2 denote the onset position and the top of the VB. (**b**) CB minimum and VB maximum with respect to the vacuum level (E_VL_) for MCN. The ionization energy IE, work function WF and electron affinity EA are indicated.

The UPS is measured using X-ray Photoelectron Spectroscopy (Japan, Axis Ultra Dld). To ensure the accuracy of experimental results, the surface of the MCN thin films is etched by Ar ion-beam for 2 min before UPS characterization to eliminate surface contamination. The band gap of MCN is determined by a Fourier Transform-Infrared Spectrometer (Germany, Bruker Vertex 80v), and is found to be about 0.69 eV. Figure 5a shows the UPS spectrum of the films with He I radiation (21.22 eV). It includes the photoemission onset at position 1, from which the vacuum level (E_VL_) of the surface can be deduced, and the density of states near the valence band edge at position 2.

Then the Fermi level (E_F_) and WF of the MCN films can be directly determined via a very standard method, i. e., WF = *hv* – (E_cutoff_ – E_F_), where *hv* is the photon energy, and E_cutoff_ is the cutoff energy and equal to 21.22 eV. Thus, the vacuum level position (E_VL_) and IE of the MCN are determined by the VB spectrum.

Combined with the band gap measured by transmission, the electronic structure of MCN thin films is determined unambiguously and are summarized in the energy diagrams of Fig. 5b with IE=4.63 eV, WF = 3.96 eV and EA = 3.94 eV. It shows that the film is indeed n-type nature, which has been confused as conducting p-type materials in the early reports^25^. The consistency of the MCN electronic structure with the levels of metal electrodes and semiconductors provides a great opportunity to optimize the energy band structure of the devices for charge injection and extraction in organic electronics and energy optoelectronics^6^,^26^,^27^.

**Figure 6:**
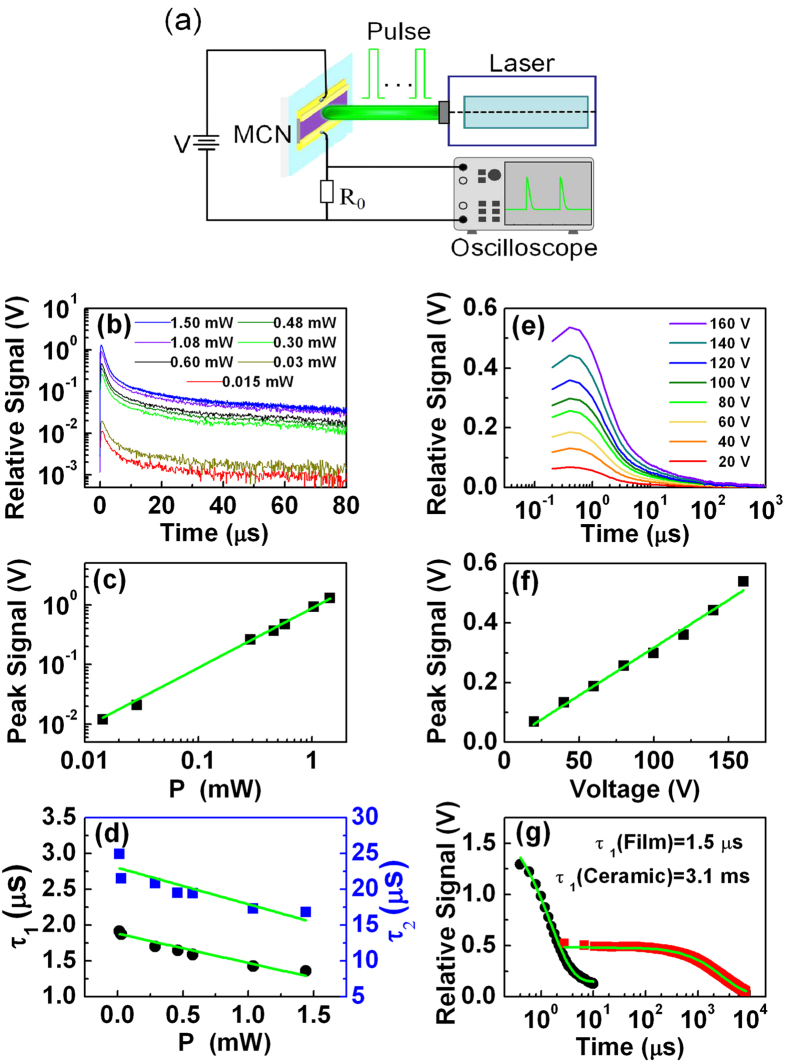
Impulse laser radiation and signal decay experiments for the MCN devices. (**a**) Experimental configuration. (**b**) Signal response radiated under different laser power. (**c**) The relation of response at peak signal versus incident power 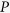 and its linear fitting. (**d**) Time constants at different laser power 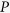 and linear fittings. (**e**) Signal response at different voltage 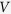 with fixed power 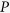 = 0.5 mW. (**f**) Peak response signal versus the biased voltage of the device and its linear fitting. (**g**) Comparison of signal decay response between the nanoscale thin films (black dots) and ceramic materials (red squares). Time constants are determined by an exponential fitting (green curves).

To further evaluate the performance of the nanofilms, we carry out photon excited carrier relaxation dynamics for the MCN materials. Figure 6a shows the configuration of the impulse experiment. A Nd:YAG nanosecond laser is used to excite nonequilibrium carriers with the wavelength of 1064 nm, the corresponding quantum energy is greater than the band gap of MCN materials. The pulse width of the laser is 7.8 ns and the repetition frequency is 10 Hz. The power incident on the devices is adjusted by optical attenuators.

Figure 6b shows the signal 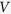 response irradiated by laser impulse with the power 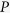 of 0.015–1.5 mW. The signal increases suddenly when the device is irradiated and decays exponentially after radiation is over. The variation of response at peak signal is linear with incident power over at least two orders (Fig. 6c), which shows a great dynamic range response. The decay of signal 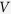 in Fig. 6b after irradiation is fitted reasonably by two time constants 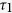 and 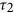 using the formula 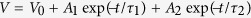 (where 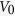 is offset of the signal, 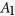 and 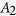 are amplitudes of the decay signals.), respectively. Figure 6d shows the results of 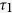 and 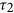 at different incident power. Usually three carrier recombination mechanisms, Auger, Shockley–Read–Hall (SRH), and radiative, determine the total recombination rate. The short time constant 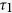 is determined by the recombination of laser generated carriers. Assuming equal electron and hole concentrations 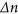 are generated by the pulsed laser, the lifetime for Auger recombination can be expressed as,


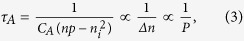


where 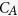 is Auger coefficient, 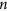 and 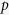 are electron and hole concentrations, 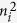 is intrinsic carrier concentration. Eq. 3 indicates that the lifetime is inversely proportional to the incident power 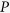, i.e. the time constant decreases as the incident power increases. A linear fitting to 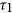 in Fig. 6d demonstrates that Auger recombination is dominant. In the Auger recombination, the excess energy given off by an electron recombining with a hole is given to a second electron. The newly excited electron then gives up its additional energy in a series of collisions with the lattice, relaxing back to the edge of the energy band. Therefore, the long time constant 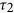 is attributed the thermal diffusion of the MCN film to the substrate. A linear fitting to 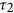 in Fig. 6d shows that it decreases as 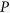 increase, similar to that of 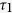.

Figure 6e shows the pumped signal response at different biased voltage with fixed power. The signal is enhanced when the biased voltage increased. The peak response signal is linear with the biased voltage, even at high voltages, up to 160 V (Fig. 6f). However, the two time constants of decay 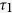 and 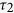, keep almost constant at different biased voltage (not shown here).

Finally, Fig. 6g compares the recombination time constant 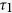 of the pumped carriers between the nanofilms and ceramic materials sintered by nitrates of Mn, Co, and Ni. It shows clearly that the time constant of the films is about 3 orders smaller than that of ceramic material, which suggests that the boundaries and defects are greatly reduced in the thin films; as the ceramic materials are composed of 1-3 micron grains with the rich defects and boundaries, which reduces the carrier recombination rate, i.e., increases the corresponding time constant^28^. Fortunately, the performance of the nanofilms is much improved; the time constant is dominated by intrinsic Auger recombination, which is essential to the development of high-speed TMO devices.

## Conclusion

In conclusion, high performance spinel MCN nanofilms with (220) preferred orientation have been fabricated via RF sputtering, with well controlled oxidation states by merging the decomposition temperatures of the precursors to the initial synthesis temperature of the films. MCN nanofilms have been realized with both moderate resistivity and large absolute TCR value simultaneously for the first time. It shows a fast intrinsic response, about 3 orders shorter than the extrinsic one of the ceramic materials, observed by the recombination of photon pumped carriers. The successful breakthrough of high performance of MCN thin films ensures the opportunity for wide ranging applications. The results are very important to improve power conversion efficiency in organic electronics and energy optoelectronics by optimizing the band structures, to manufacture broadband focal plane arrays for advanced optical detection with long-term stability, and to develop high-speed TMO devices in next generation of oxide electronics and optoelectronics.

### Experimental

Thin films of MCN with nominal composition Mn_1.4_Co_1.0_Ni_0.6_O_4_ were grown starting from pressed powder of MCN target material, which was obtained by drying the chemical solution of Mn, Co, Ni acetates of Mn(CH_3_COO)_2_·4H_2_O, Co(CH_3_COO)_2_·4H_2_O, Ni(CH_3_COO)_2_·4H_2_O with an atomic ratio of 7:5:3 (the target ratio) and calcined at 850 °C. Then a polycrystalline MCN ceramic wafer (Φ 60 mm × 4 mm) with a cubic spinel phase and no prefer orientation, was prepared via traditional sintering method. Prior to film deposition, the base pressure was evacuated to approximately 5 × 10^−3^ Pa, and a working pressure of 0.4 Pa. High purity (>99.99%) argon was applied to provide the plasma. Al_2_O_3_ substrates were annealed at 750 °C for ~3 min before growth. The growth temperature was set 200 °C. MCN film sample was deposited under 100 W of RF power for 1.5 hours and post annealed at 750 °C for 90 min for crystallization. MCN bolometer was mounted to a conductive heat-sink sealed in a ceramic package. Electrical properties were measured by a Keithley 2400 and associated temperature control systems. The MCN ceramic material in the experiment was manufactured by the typical traditional method, calcined at 1100 ~ 1200 °C for 30 min and cooled to room temperature naturally. The material is polycrystalline, with a distribution of grains sizes from 1 to 3 micron, determined from SEM images. The precursor was manufactured from nitrates of Mn, Co, Ni in chemical solution of the target ratio.

## Additional Information

**How to cite this article**: Huang, Z. *et al.* High performance of Mn-Co-Ni-O spinel nanofilms sputtered from acetate precursors. *Sci. Rep.* **5**, 10899; doi: 10.1038/srep10899 (2015).

## Supplementary Material

Supplementary Information

## Figures and Tables

**Figure 1:**
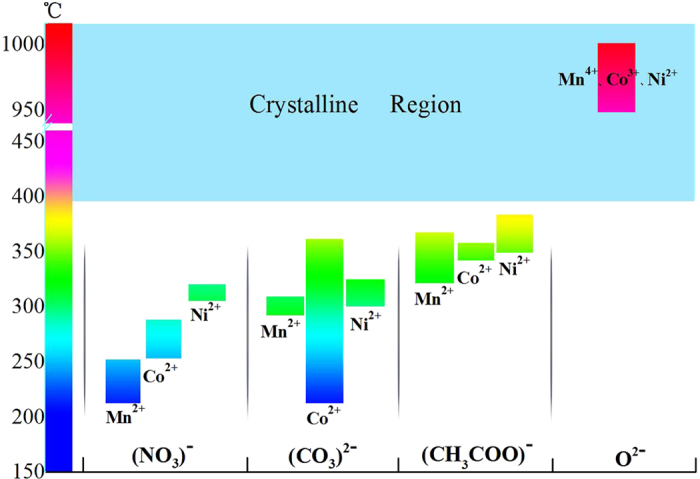
Schematic illustration of the MCN crystalline temperature and the decomposition temperatures of different raw precusor materials of nitrates, carbonates, acetates and oxides, respectively. The decomposition temperatures of acetates are followed immediately by the crystalline temperature of MCN thin films beginning at ~400 °C. The cations and anions are labeled separately for a clear show. The crystalline region (>400 °C) is highlighted by light blue color. The colorized columnar indicates the temperatures which vary from 150 °C to 450 °C and over 950 °C. A break to minish the columnar length is inserted.
