# Supplementary material for: High performance of Mn-Co-Ni-O spinel nanofilms sputtered from acetate precursors
Source: Sci Rep. 2015 Jun 8;5:10899. doi: 10.1038/srep10899 (PMC4458888; doi:10.1038/srep10899)
Supplement: Supplementary Information [file srep10899-s1.doc]

Supporting Information

High performance of Mn-Co-Ni-O spinel nanofilms sputtered from acetate precursors

*Zhiming Huang*^a,b^,**, Wei Zhou*^a,b^*, Cheng Ouyang*^a^*, Jing Wu*^a^*, Fei Zhang*^a^*, Jingguo Huang*^a^*, Yanqing Gao*^a^*, and Junhao Chu*^a^

^a^National Laboratory for Infrared Physics, Shanghai Institute of Technical Physics, Chinese Academy of Sciences, 500 Yu Tian Road, Shanghai 200083, Peoples Republic of China

^b^Key laboratory of Space Active Opto-Electronics Technology, Shanghai Institute of Technical Physics, Chinese Academy of Sciences, 500 Yu Tian Road, Shanghai 200083, Peoples Republic of China

***E-mail: zmhuang@mail.sitp.ac.cn

**Thermal decomposition process of different precursors for Mn-Co-Ni-O target materials fabrication**

Here we list the decomposition temperature of each reactive material for four groups of typical precursors to fabricate Mn-Co-Ni-O (MCN) target materials in **Table S1**. (1) For the group of nitrates of Mn, Co and Ni, the initial decomposition temperature begins at about 220 °C, 250 °C and 310 °C for Mn(NO_3_)_2_, Co(NO_3_)_2_ and Ni(NO_3_)_2_, respectively, and the decomposition temperature spans 30 °C for each decomposition process. Next, MnO_2_, Co_3_O_4_ and NiO are produced after the decomposition process. (2) For the group of carbonates of Mn, Co and Ni, the initial decomposition temperature begins at about 300 °C, 220 °C, and 300 °C for MnCO_3_, CoCO_3_ and NiCO_3_, respectively, and the span of the decomposition temperature is about 10-20 °C for MnCO_3_ and NiCO_3_, while 140 °C for CoCO_3_. (3) For the group of acetates of Mn, Co and Ni, the initial decomposition temperature begins at about 320 °C, 350 °C and 350 °C for Mn(CH_3_COO)_2_, Co(CH_3_COO)_2_, and Ni(CH_3_COO)_2_, respectively, and the decomposition temperature spans about 30-50 °C for each decomposition process. (4) For the group of Mn_3_O_4_、Co_3_O_4_ and NiO, the compounds crystallize at a very high of temperature above 950 °C.

**Table S1.** Decomposition temperatures of various reactive materials taken from the corresponding references.

| **Reactive materials** | **Decomposition temperature range (°C)** | **Reference** |
| --- | --- | --- |
| Mn(NO3 )2 | 220～250 | S1 |
| Co(NO3 )2 | 250～280 | S2 |
| Ni(NO3)2 | Around 310 | S3 |
| MnCO3 | Around 300 | S3 |
| CoCO3 | 220～360 | S2 |
| NiCO3 | 300～320 | S4 |
| Mn(CH3COO)2 | 320～370 | S5 |
| Co(CH3COO)2 | Around 350 | S2 |
| Ni(CH3COO)2 | 350～380 | S2 |
| Mn3O4 | 950～1000 | S6 |
| Co3O4 | 950～1000 | S2 |
| NiO | 950～1000 | S7 |

**Manganese ion distributions in Mn_2-_*_x_*Co_2_*_x_*Ni_1-_*_x_*O_4_ (*x* = 0.4, 0.5, 0.6, 0.8) series films**

In the main text, we select the composition of Mn_1.4_Co_1.0_Ni_0.6_O_4_ because it is an optimized oxidizing status with the composition of Mn^3+^ matches well with that of Mn^4+^. To show this is true case, we have measured the manganese ion distributions in Mn_2-x_Co_2x_Ni_1-x_O_4_ (x = 0.4, 0.5, 0.6, 0.8) series films.


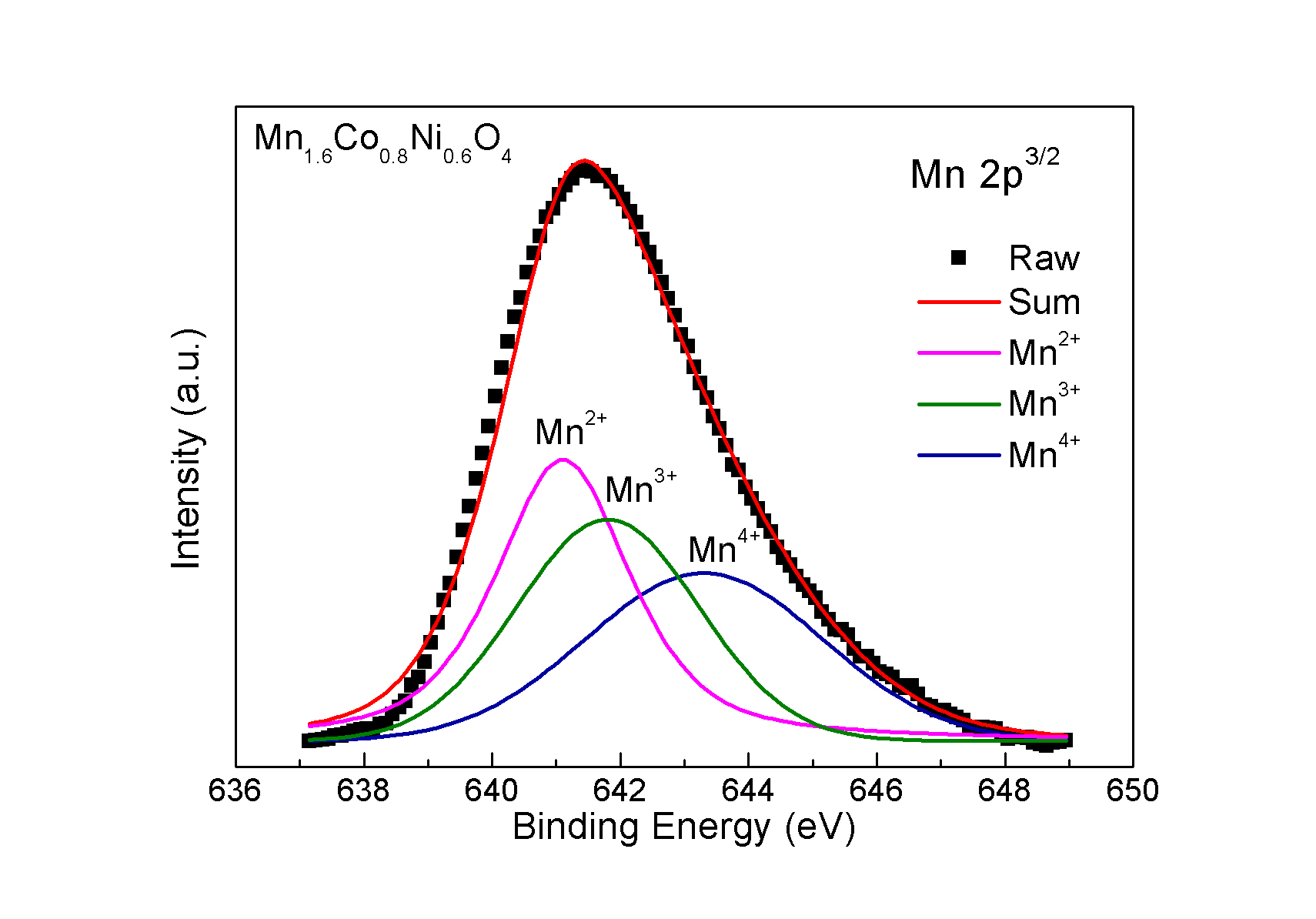

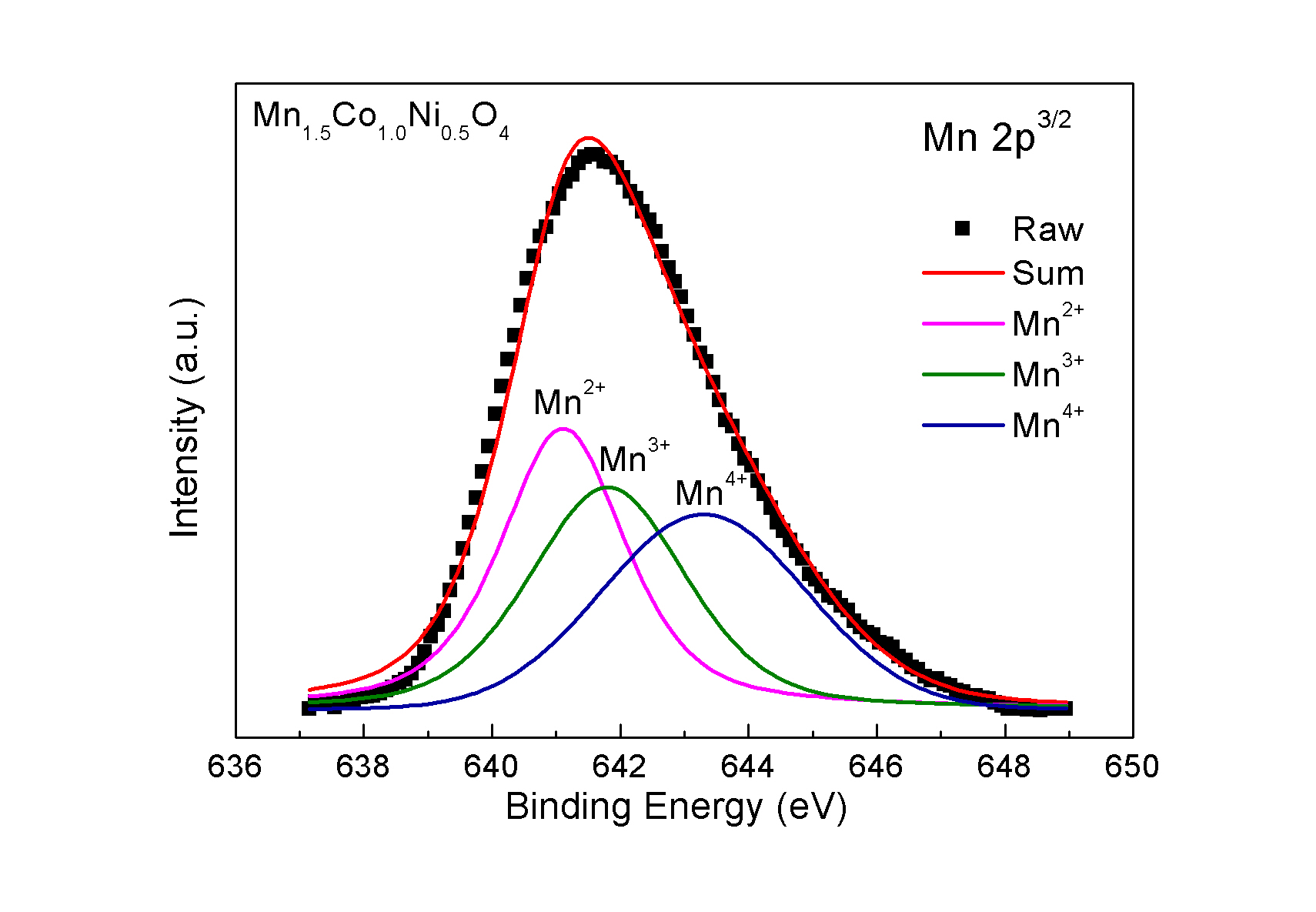


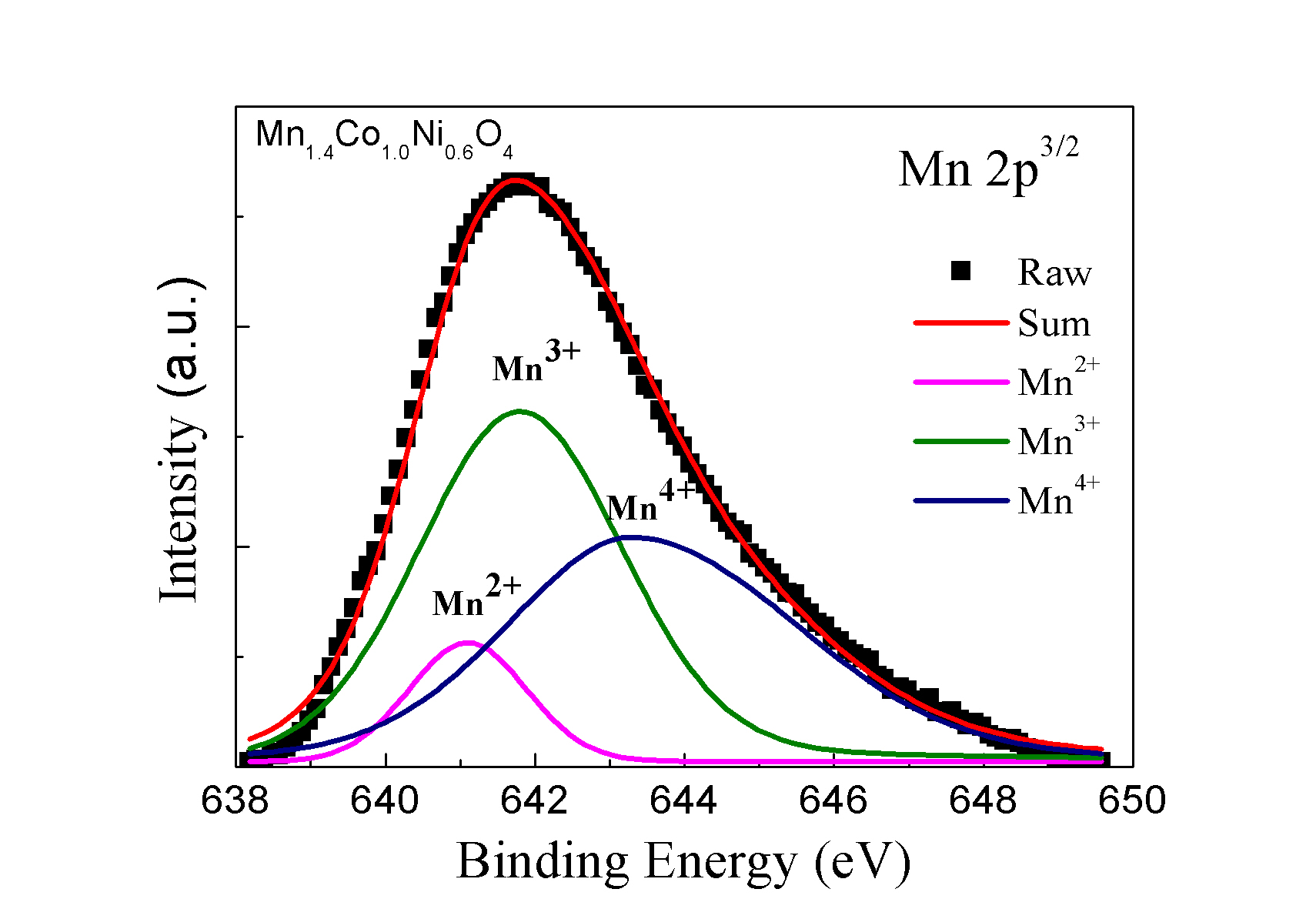

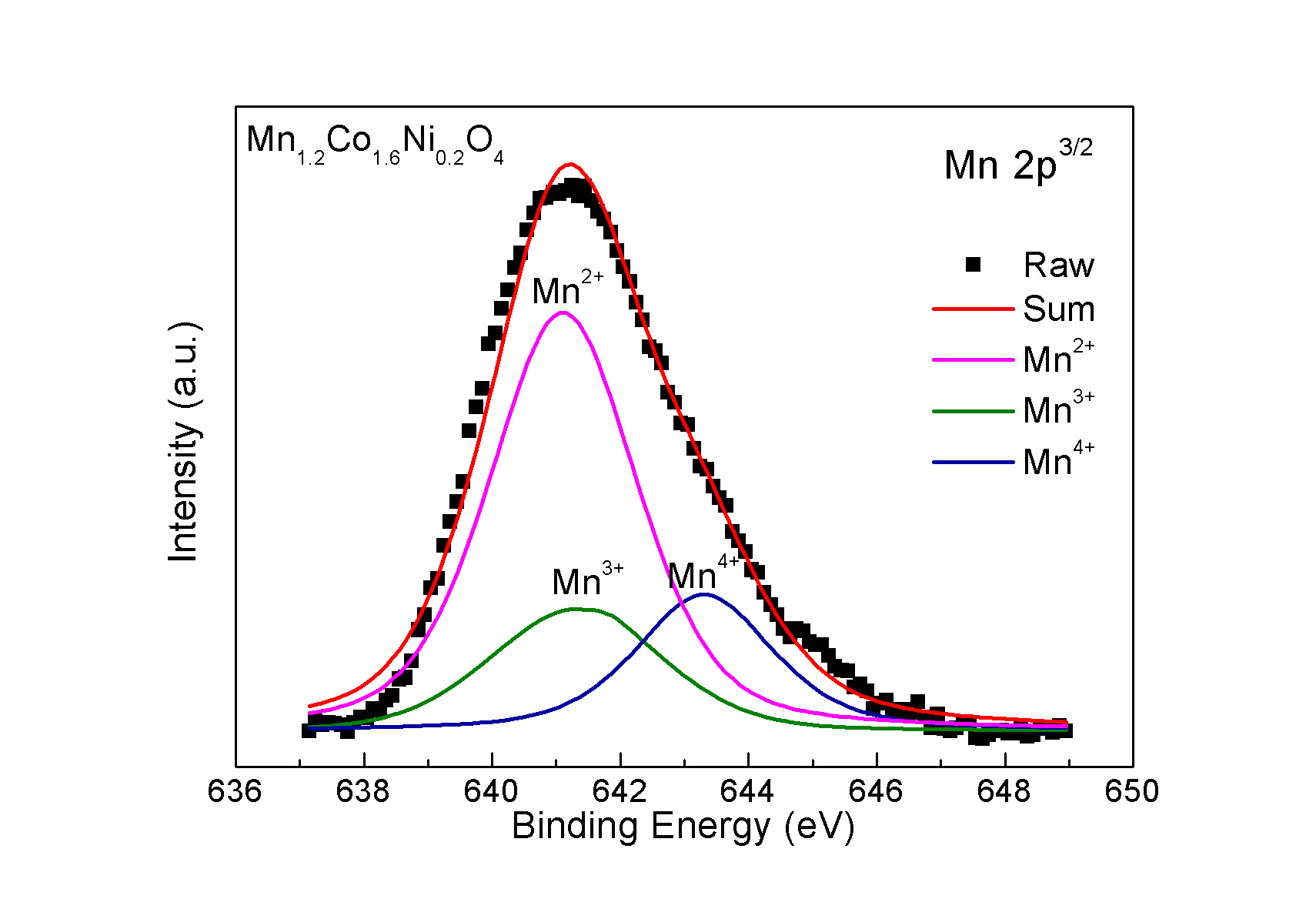


**Figure S1.** Mn 2p^3/2^ XPS spectra and deconvoluted curves for Mn^2+^, Mn^3+^ and Mn^4+^ after background subtraction in the Mn_1.6_Co_0.8_Ni_0.6_O_4_, Mn_1.5_Co_1.0_Ni_0.5_O_4_, Mn_1.4_Co_1.0_Ni_0.6_O_4_, and Mn_1.2_Co_1.6_Ni_0.2_O_4_ nanofilms.

The XPS spectra for Mn 2p^3/2^ signals of the Mn_2-_*_x_*Co_2_*_x_*Ni_1-_*_x_*O_4_ (*x* = 0.4, 0.5, 0.6, 0.8) series films are deconvoluted after background subtraction by a fitting process using XPS standard software. We obtain the amount of Mn^2+^, Mn^3+^ and Mn^4+^ ions in the Mn_1.6_Co_0.8_Ni_0.6_O_4_,  Mn_1.5_Co_1.0_Ni_0.5_O_4_, Mn_1.4_Co_1.0_Ni_0.6_O_4_, and Mn_1.2_Co_1.6_Ni_0.2_O_4_ nanofilms, according to the XPS spectral analysis for Mn 2p^3/2^ signals. The binding energies and FWHM values of Mn^2+^, Mn^3+^ and Mn^4+^ are referenced by MnO, ZnMn_2_O_4_ and MgNiMnO_4_ compounds, respectively. The results of fitting are displayed in **Figure S1**.

The derived ratios of the amounts of Mn^2+^, Mn^3+^ and Mn^4+^ ions are listed as follows:

Mn_1.6_Co_0.8_Ni_0.6_O_4_: [Mn^2+^]:[Mn^3+^]:[Mn^4+^] = 0.62:0.43:0.55;

Mn_1.5_Co_1.0_Ni_0.5_O_4_: [Mn^2+^]:[Mn^3+^]:[Mn^4+^] = 0.422:0.605:0.473;

Mn_1.4_Co_1.0_Ni_0.6_O_4_: [Mn^2+^]:[Mn^3+^]:[Mn^4+^] = 0.118:0.641:0.641;

Mn_1.2_Co_1.6_Ni_0.2_O_4_: [Mn^2+^]:[Mn^3+^]:[Mn^4+^] = 0.802:0.245:0.153;

From the above results, we can find only the ratio of Mn^3+^ to Mn^4+^ is 1:1 in the Mn_1.4_Co_1.0_Ni_0.6_O_4_ nanofilms, which is the optimized oxidizing status as indicated by Eq. 2 in the main text.

**Characterization on the performance for IR detection**

The detection ability for IR bolometer can be expressed as *|*TCR*|*/(*γ/n*)^0.5^ in terms of Hooge’s relation, where both high TCR and low *γ/n* are favorable.^S8^ In polycrystalline like MCN film, the TCR value and the resistance are largely influenced by the grains and the grain boundaries.^S9^ For example, grain boundaries may introduce extrinsic conduction mechanism, which might increase excess noise (or *1/f* noise). Therefore, it is important to improve the performance of the MCN films with low *1/f* noise and good electrical properties. The IR bolometer based on our highly oriented MCN films shows a high TCR value of -3.9% K^-1^ and low excess noise (normalized value *γ/n* of 1.0×10^-21^ cm^3^) at 295 K.

Infrared bolometer based on the MCN thin films has been manufactured, with a device size of 0.12 mm×4.80 mm. A thermistor bolometer bridge circuit is applied at room temperature as shown in the inset of **Figure S2a**. The resistance of the detection thermistor strip *R_d_* is equal to that of the compensated one *R_c_*. Then the reduced resistance *R* = *R_c_·R_d_ / (R_c_+R_d_)* = 0.5 *R_d_*. The detection element is black coated, with an average absorptivity *η* of about 90 % in the range of 2 - 14 μm. Voltage fluctuations across the bolometer are amplified by ultralow noise, high impedance, FET operational amplifier with a short junction noise of 5 nV/Hz^0.5^.

**Figure S2a** shows the spectral power density of the *1/f* noise (*S_V_*) in the frequency *1/f* range of 4 - 40 Hz. Two major components are responsible for the noise spectrum: Johnson noise and excess noise, the voltage fluctuation spectral density *N_V_* can be written as:

$N_{\text{v}}=\sqrt{4\text{k}_{B}TR+S_{\text{v}}}, S_{\text{v}}=\frac{\gamma}{n}\frac{I_{b}^{2}\cdot R^{2}}{\Omega\cdot f}$, (S1)

where *k_B_* is Boltzmann constant, Ω is the sample volume, and *I_b_* is the biased current of the devices. *N_v_* has *1/f* behavior at low frequency and experiences crossover from *1/f* to white Johnson noise as *f* increases. The excess noise is dominant at low frequencies for the MCN films. By applying **Eq. S1**, the *γ/n* value is calculated to be ~ 1.0×10^-21^ cm^-3^, which is 1-2 orders lower than that of α-Si:H^S12^ and VO_2_ films^S13^.

**Figure S2.** (a) Noise spectrum for the MCN thin film detector in the range of 4 - 40 Hz. Red line: linear fitting to the noise spectrum. Inset: experimental setup for noise measurement. (b) Frequency-dependent responsivity and detectivity of the MCN detector operated at ± 20 V bias.

Next, the specifications of the bolometer can be calculated: the responsivity R_v_ is 36 V/W and the detectivity *D^*^* is 0.5×10^7^ cm·Hz^0.5^/W at the frequency of 11 Hz. By fitting the R_v_ - *f* relationship in Figure S2b with:

$\text{R}_{\text{v}}=I_{b}\frac{dR}{dT}\frac{\eta}{G\sqrt{1+(2\pi f\tau)^{2}}}=I_{b}\frac{dR}{dT}\frac{A}{\sqrt{1+(2\pi f\tau)^{2}}}$, (S2)

where the time constant $\tau$is about 16 ms and the absorptance$\eta$=0.9. The coefficient *A* can be deduced at the same time. Then thermal conductance of the device *G* is estimated: *G* = *η/A* ≈ 1.2×10^-2^ W/K.

For an infrared (IR) detector without resonant cavity or absorbed layers, the detectivity at low frequency regime ($S_{\text{v}}$>>$4k_{B}TR$) can be written as:

$D^{*}\approx\frac{\left|TCR\right|\cdot V\cdot(1-e^{-\alpha d})}{2G\sqrt{1+\omega^{2}\tau^{2}}}\bullet\frac{\sqrt{\text{S}}}{\sqrt{\gamma/n}\frac{I\cdot R}{\sqrt{v\cdot f}}}\approx\frac{Q_{m}\cdot d^{1.5}\cdot\alpha\cdot d_{0}\cdot f^{0.5}}{2k_{0}\sqrt{1+4\pi^{2}f^{2}\tau^{2}}},$ (S3)

where *d*_0_ represents the thickness of the substrate; *k*_0_ represents the heat conductivity of the substrate and *G = k*_0_*S/d*_0_ with sample area *S*. Here we use the approximation: (1-*e*^-^*^αd^*) ≈ *αd*, since for transition metal oxide films with a thickness of about 0.1-1 μm, the absorption generally ranks about less than 20 % in IR region. The figure of merit for IR detection materials can be defined as: $Q_{m}=\left|TCR\right|/(\gamma/n)^{0.5}$. The calculated *Q_m_* factor is about 1.2×10^11^ %K^-1^cm^-1.5^ for the MCN films, which is higher than that of typical α-Si films (5×10^9^ %K^-1^cm^-1.5^) and VO_2_ (1×10^10^ %K^-1^cm^-1.5^) films.^S11^

It is of interest to estimate the detection ability of the MCN films based micro bolometer. By applying the relationship *d*=*λ/4n*, (where the wavelength *λ*=12 μm and refractive index n≈2), and assumed *η*= 0.9, *G*=10^-6^ W/K, *τ*=*C/G*≈5 ms, consider a MCN film bolometer sized 50×50×1.5 μm^3^, we estimate the responsivity R_v_=1.6×10^4^ V/W, *D**≈0.5×10^9^ cm·Hz^1/2^W^-1^ at 30 Hz. The expected performance of the MCN detectors is superb compared to other thermal detectors reported previously. ^S14,S15^

**Comparison of the recombination time between MCN thick film and ceramic material**

In order to demonstrate that the recombination time constant $\tau_{1}$ of MCN nanofilm device is an intrinsic mechanism, which is independent on the thickness of the films, we have carried out the impulse experiment to a thick film with 10 µm and compared $\tau_{1}$ of the film to that of the ceramic material with the same device size of 0.12 mm × 0.18 mm and the same thickness of 10 µm. The decay of signal *V* after irradiation is fitted reasonably by two time constants $\tau_{1}$ and $\tau_{2}$ using the formula $V=V_{0}+A_{1}\exp(-t/\tau_{1})+A_{2}\exp(-t/\tau_{2})$ (where $V_{0}$ is the offset of the signal, and $A_{1}$ *a*nd $A_{2}$ are the amplitudes of the decay.) The fitting results are shown in **Figure S3**. It can be seen that the recombination time constant $\tau_{1}$ of MCN film device with 10 µm thickness is approximately equal to that of the MCN nanofilm device with 170 nm thickness. That is to say, $\tau_{1}$ of the thick film is also about 3 orders smaller than that of ceramic material, which demonstrates that the recombination time is an intrinsic mechanism, which is not related to the size in thickness. **Figure S4** shows the SEM microscopy of MCN thick film and ceramic material. We can see that the ceramic material is composed of lots of grains, but the film is much dense. The porosity and grain boundaries in the ceramic material are highly greater than that in the thick film. The great descrease of the recombination time in the film can be attributed to the much reduction in the density of boundaries and defects in the film, compared to the that of the ceramic material.


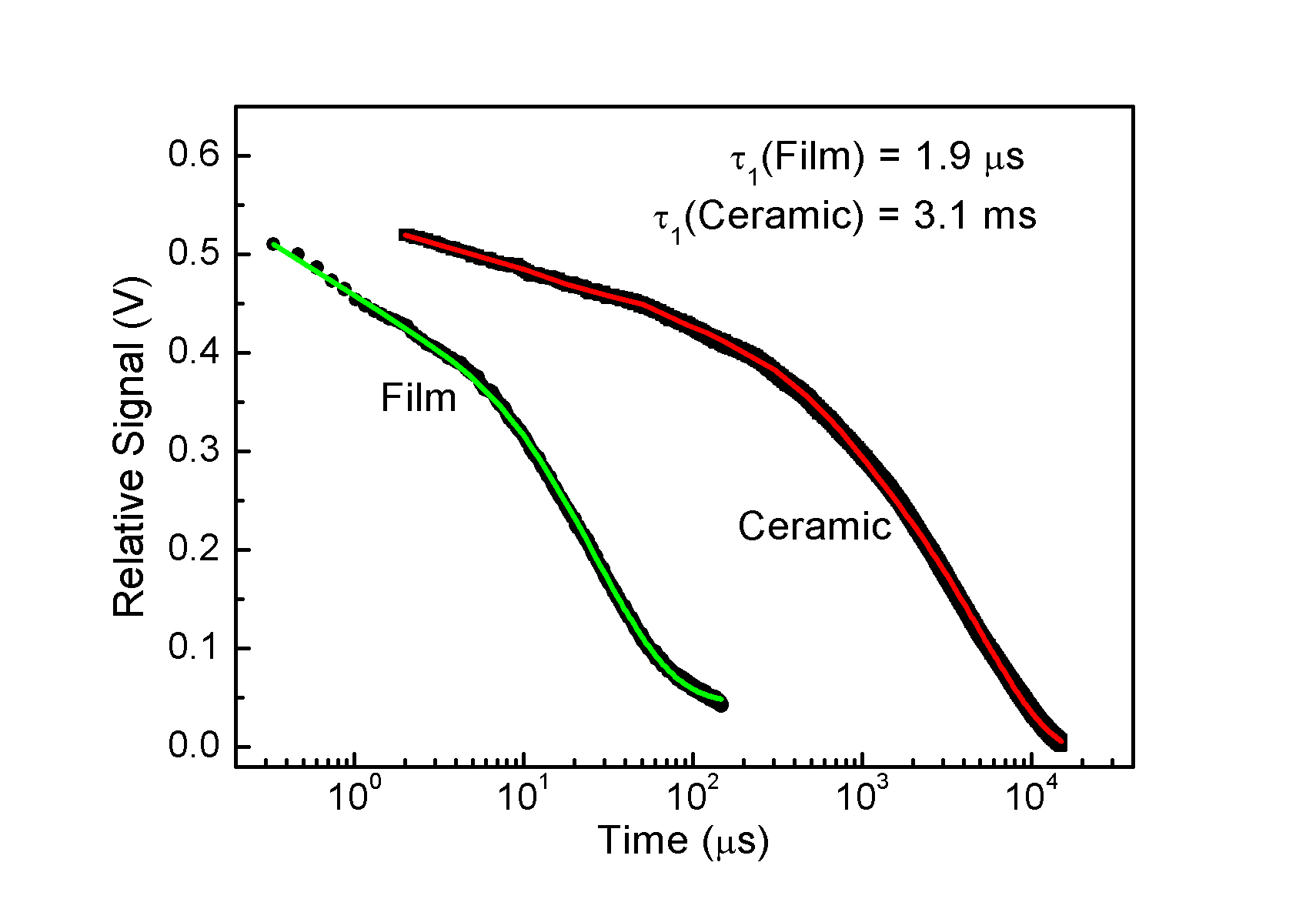


**Figure S3.** Comparison of signal decay response between the MCN thick film and ceramic material devices with same size of 0.12 × 0.18 mm and thickness of 10 µm under 0.5 mW laser power and 40 V bias voltage.


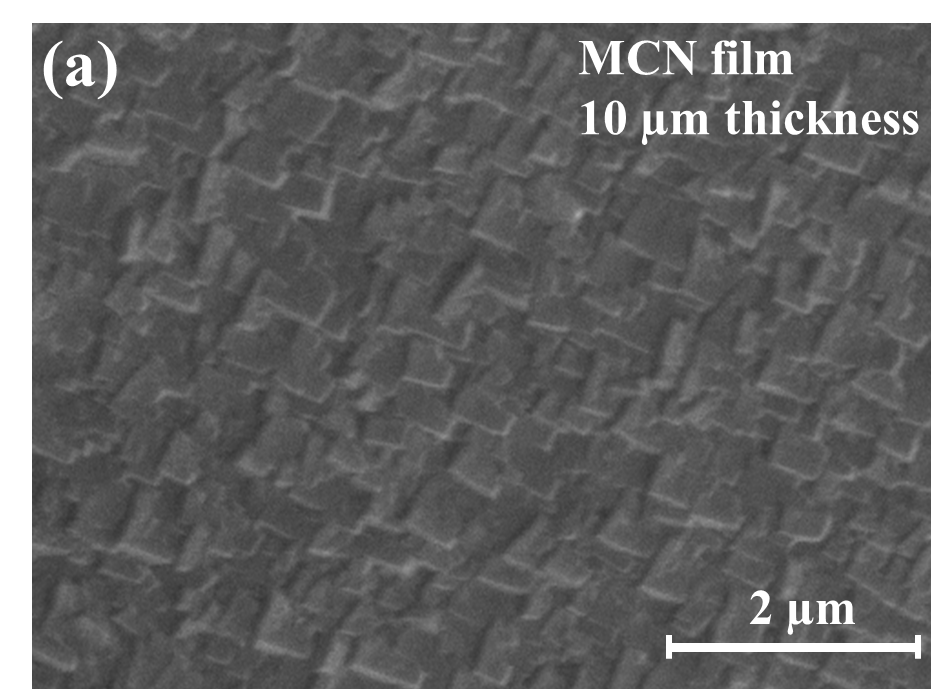

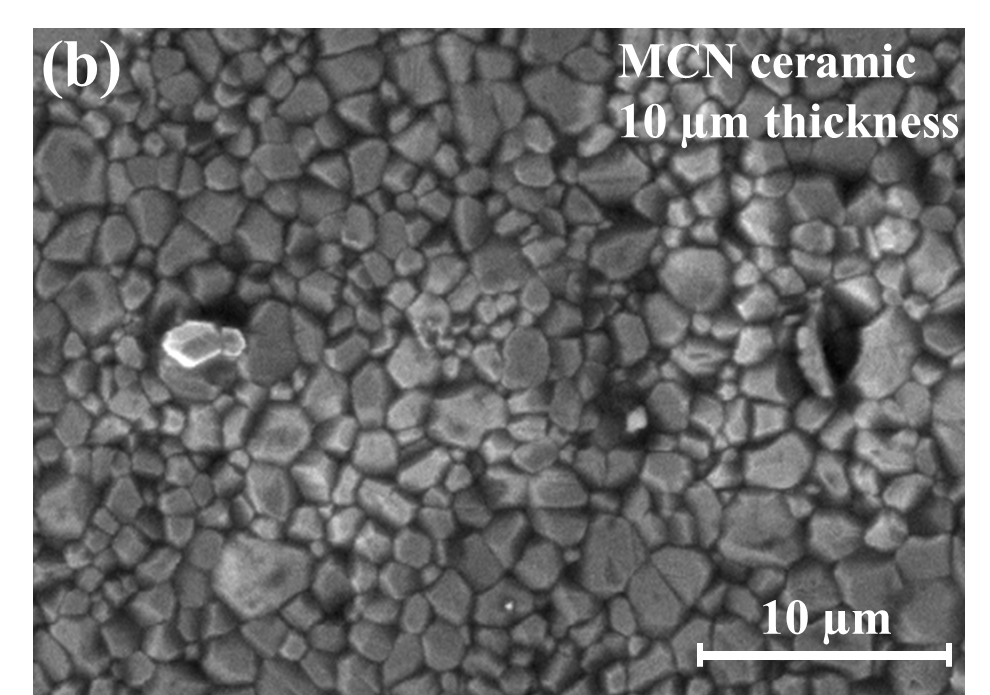


**Figure S4.** The SEM microscopy pictures of MCN thick film and ceramic material.

**References.**

S1. Jian, G. Understanding and Tuning Nanostructured Materials for Chemical Energy Conversion, University of Maryland, 2014.

S2. Liptay, G. Atlas of Thermoanalytical Curves: (TG-, DTG-, DTA-curves Measured Simultaneously), Heyden and Son,1977.

S3. Shaheen, W. M. & Selim, M. M. Thermal behaviour of pure and mixed ammonium, molybdate tetrahydrate and aluminum hydroxide *AFINIDAD.* Thermochim Acta.**322**, 117 (1998).

S4. Zhan, Y. J., Yin, C. R., Zheng, C. L., Wang, W. Z. & Wang, G. H. J. A simple method to synthesize NiO fibers. Solid State Chem. **177**, 2281 (2004).

S5. Shao, C. L., Guan, H. Y., Liu, Y. C., Li, X. L. & Yang, X. H. J. Preparation of Mn_2_O_3_ and Mn_3_O_4_ Nanofibers via an Electrospinning Technique. Solid State Chem. **177**, 2628 (2004).

S6. Moon, J., Awano, M., Takai, H. & Fujishiro, Y. J. Synthesis of nanocrystalline manganese oxide powders: Influence of hydrogen peroxide on particle characteristics. Mater. Res. **14**, 4594 (1999).

S7. Marinšek, M., Marinšek, K. & Maček, J. J. Preparation of Ni–YSZ composite materials for solid oxide fuel cell anodes by the gel-precipitation method. Power Sources. **86**, 383 (2000).

S8. Dutta, P., Horn, P. M. Low-frequency fluctuations in solids: 1/*f* noise. Rev. Mod. Phys. **53**, 497 (1981).

S9. He, L. & Ling, Z. Y. Studies of temperature dependent ac impedance of a negative temperature coefficient Mn-Co-Ni-O thin film thermistor. Appl. Phys. Lett. **98**, 242112 (2011).

S10. He, L. & Ling, Z. Y., J. Electrical conduction of intrinsic grain and grain boundary in Mn-Co-Ni-O thin film thermistors: Grain size influence. Appl. Phys. **110**, 093708 (2011).

S11. Lisauskas, A., Khartsev, S. I. & Grishin, A. Tailoring the colossal magnetoresistivity: La_0.7_ (Pb_0.63_ Sr_0.37_)_0.3_ MnO_3_ thin-film uncooled bolometer. Appl. Phys. Lett. **77**, 756 ( 2000).

S12. Zerov, V. Y. & Malyarov, V. G. J. Heat-sensitive materials for uncooled microbolometer arrays. Opt. Technology. **68**, 939 (2001).

S13. Zerov, V. Y. et al. Vanadium oxide films with improved characteristics for IR microbolometric matrices. Tech. Phys. Lett. **27**, 378 (2001).

S14. Chen, C. H., Yi, X. J., Zhao, X. R. & Xiong, B. F. Characterizations of VO2-based uncooled microbolometer linear array. Sensor Actuat. A-Phys. **90**, 212 (2001).

S15. Choudhary, R. J. et al. Evaluation of manganite films on silicon for uncooled bolometric applications. Appl. Phys. Lett. **84**, 3846 (2004).
